# Prospective Study on the Association between Harm Avoidance and Postpartum Depressive State in a Maternal Cohort of Japanese Women

**DOI:** 10.1371/journal.pone.0034725

**Published:** 2012-04-10

**Authors:** Kaori Furumura, Takayoshi Koide, Takashi Okada, Satomi Murase, Branko Aleksic, Norika Hayakawa, Tomoko Shiino, Yukako Nakamura, Ai Tamaji, Naoko Ishikawa, Harue Ohoka, Hinako Usui, Naomi Banno, Tokiko Morita, Setsuko Goto, Atsuko Kanai, Tomoko Masuda, Norio Ozaki

**Affiliations:** 1 Department of Psychiatry, Nagoya University Graduate School of Medicine, Nagoya, Aichi, Japan; 2 Faculty of Policy Studies, Nanzan University, Seto, Aichi, Japan; 3 Department of Nursing, Sugiyama Jogakuen University, Nagoya, Aichi, Japan; 4 Graduate School of Education and Human Development, Nagoya University, Nagoya, Aichi, Japan; 5 Graduate School of Law, Nagoya University, Nagoya, Aichi, Japan; Catholic University of Sacred Heart of Rome, Italy

## Abstract

**Background:**

Recent studies have displayed increased interest in examining the relationship between personality traits and the onset, treatment response patterns, and relapse of depression. This study aimed to examine whether or not harm avoidance (HA) was a risk factor for postpartum depression measured by the Edinburgh Postnatal Depression Scale (EPDS) and the state dependency of HA.

**Methods:**

Pregnant women (n=460; mean age 31.9±4.2 years) who participated in a prenatal program completed the EPDS as a measure of depressive state and the Temperament and Character Inventory (TCI) as a measure of HA during three periods: early pregnancy (T1), late pregnancy (around 36 weeks), and 1 month postpartum (T2). Changes in EPDS and HA scores from T1 to T2 were compared between the non depressive (ND) group and the postpartum depressive (PD) group.

**Results:**

There was no significant difference in the level of HA between the ND and PD groups at T1. In the ND group, EPDS and HA scores did not change significantly from T1 to T2. In the PD group, both scores increased significantly from T1 to T2 (EPDS, p<0.0001; HA, p<0.048). In the ND and PD groups, a significant positive correlation was observed in changes in EPDS and HA scores from T1 to T2 (r=0.31, p=0.002).

**Conclusions:**

These results suggest that HA cannot be considered a risk factor for the development of postpartum depression measured by EPDS. Furthermore, HA may be state dependent.

## Introduction

Recent studies have displayed increased interest in examining the relationship between personality traits and the onset, treatment response patterns, and relapse of depression [1], [2], [3]. Personality traits have been demonstrated to play an important role in the onset of major depression, and have been considered useful as a possible outcome for the prevention and early detection of and intervention for symptoms of depression. A recent study demonstrated that psychosocial adversity interacts with neuroticism in the etiology of major depression, and the impact of neuroticism on illness risk is greater at high than at low levels of adversity [4].

On the other hand, previous studies have examined the relationship between depression and personality as well as changes in personality traits due to the onset of depression and have demonstrated a significant association [5], [6], [7], [8]. In a previous study, our colleagues examined the state dependency of the Temperament and Character Inventory (TCI) [9] in patients with major depression [7] and reported that harm avoidance (HA), an anxiety-related trait associated with neuroticism, decreased as symptoms of depression improved in patients with major depressive disorder (MDD). However, these patients were medicated with antidepressants; therefore, there may be state dependency of personality traits regarding depression [7].

Most previous studies that have explored the relationship between personality traits and depression have done so after the onset of depression or when medical intervention was already underway. To date, only a handful of studies have examined the association between personality and symptoms of depression before the onset of depression. Thus, studies examining the relationship between personality and the development of depression within a prospective cohort design should consider the following three points: 1) the longitudinal changes in personality traits through measured assessments or observations; 2) the effects of changes in depression or symptoms of depression within a continuous spectrum in order to capture individuals that fall underneath beneath the threshold); and 3) the heterogeneity in the pathophysiology of depression within individuals diagnosed with having MDD.

However, previous prospective cohort studies that examined the relationship between personality and depression did not take into account the instability of personality within a longitudinal timeframe, and assessed depression using a categorical (mental disorder present or not present) approach. Additionally, previous studies did not explore the heterogeneity found in participants diagnosed with MDD, for example, by examining qualitative differences within individuals diagnosed with MDD.

Postpartum depression is a specific type of depression used to describe a continuum of depressive symptoms and diagnosis that occur from several weeks to several months after childbirth. The operational definition given in the *Diagnostic and Statistical Manual of Mental Disorders*, *Fourth Edition Text Revision* uses a time frame of 4 weeks after childbirth for the onset of symptoms. We previously conducted a prospective study on a maternal cohort of Japanese women using a longitudinal design and a questionnaire to elucidate the prevalence of postpartum depression (measured by the EPDS), changes in depression symptoms, and biopsychosocial factors affecting the onset of depressive state during pregnancy and postpartum period [10].

The present study using a maternal cohort of pregnant Japanese women is suitable to study personality traits associated with the onset of depressive symptoms for the following three reasons: 1) we can evaluate whether or not participants had postpartum depression within a relatively short period (about 1 year) because pregnant women are susceptible to the development of postpartum depression and the incidence of postpartum depression is high (approximately 10–15%) [11], [12], [13], [14], [15], [16], [17]; 2) we can investigate how changes in depressive symptoms in unaffected participants affects personality traits because using prospective design we can detect depressive symptoms and personality traits before onset of depression; and 3) in our cohort, subjects who presented with depressive symptoms were more homogeneous than MDD patients from previous studies because life events influencing the onset of depression included common biopsychosocial events (pregnancy and childbirth). To the best of our knowledge, there have been no prospective cohort studies that have investigated the role of personality in the onset of postpartum depression.

Thus, the present study aimed to examine the relationship between personality and depressive state to elucidate 1) whether HA was a risk factor for postpartum depression and 2) whether or not mean levels in HA changed before and after the onset of depressive state, that is, to investigate the state dependency of HA.

## Results

Participant profiles are shown in Table 1. No significant differences in age were found across the four groups (p=0.81, ANOVA). There was no significant difference in HA during T1 (early pregnancy) between the ND and PD groups (Table 2). Changes in EPDS from T1 to T2 are shown in Table 3 and Figure S1. In the PD group, EPDS score increased significantly from T1 to T2. Changes in HA from T1 to T2 are shown in Table 4 and Figure S2. In the PD group, HA score also increased significantly from T1 to T2. The effect size of the HA change was smaller than the EPDS change. Correlations between changes in scores on the EPDS and HA are shown in Table 5 and Figure S3. Correlations between changes in scores on the EPDS and HA were significant (r=0.31, p=0.002). Regarding the HA subscales, only changes in fatigability and asthenia was significantly correlated with changes in EPDS scores (r=0.29, p=0.003) (Table 5).

**Table 1:** Participant profiles in the four groups.

	period						
	pregnancy		postpartum					
groups	T1	late	T2	n	%	age		p-value^a^
						mean	SD	
Non depressive (ND) group	−	−	−	331	72.0	31.9	4.1	0.81
Postpartum depressive (PD) group	−	−	+	48	10.4	32.0	3.9	
Temporary gestational depressive (TG) group	+	+	−	52	11.3	31.8	5.1	
	+	−	−					
	−	+	−					
Continuous depressive (CD) group	+	+	+	29	6.3	31.1	4.7	
	+	−	+					
	−	+	+					
All				460	100.0	31.9	4.2	

+: EPDS>8, −: EPDS<9.

T1: early pregnancy (before 25 weeks).

late: late pregnancy (around 36 weeks).

T2: postpartum (1 month).

a : ANOVA was used to test the mean differences of age within the four groups.

**Table 2:** HA scores in the ND and PD groups at T1.

	n	mean	SD	p-value^a^
ND group	331	10.2	4.5	0.60
PD group	48	11.0	4.6	
All	379	10.3	4.5	

ND group: non depressive group.

PD group: postpartum depressive group.

SD: standard deviation.

a : Student's t-test was conducted in HA scores between the ND and PD groups at T1.

**Table 3:** EPDS scores in the ND and PD groups.

		T1		T2		p-value^a^	Cohen's d
	n	mean	SD	mean	SD		
ND group	331	2.8	2.4	2.6	2.3	0.27	−0.09
PD group	48	4.0	2.2	12.2	3.4	**<0.0001**	2.86
All	379	2.9	2.4	3.8	4.0		

EPDS: Edinburgh Postnatal Depression Scale.

T1: early pregnancy (before 25 weeks).

T2: postpartum (1 month).

ND group: non depressive group.

PD group: postpartum depressive group.

SD: standard deviation.

a : Paired t-test in EPDS between T1 and T2 in the ND and PD groups.

**Table 4:** HA scores in the ND and PD groups.

		T1		T2		p-value^a^	Cohen's d
	n	mean	SD	mean	SD		
ND group	81	10.4	4.3	10.1	3.8	0.27	−0.07
PD group	18	11.1	5.0	12.3	4.2	**0.048**	0.26
All	99	10.6	4.4	10.5	3.9		

HA: harm avoidance.

T1: early pregnancy (before 25 weeks).

T2: postpartum (1 month).

a : Paired t-test in HA scores between T1 and T2 in the ND and PD groups.

**Table 5:** Correlations between HA and EPDS score changes from T1 to T2 in the ND and PD groups.

	ND and PD groups (n=99)	r	p-value
HA total		0.31	**0.002**
HA subscale	Anticipatory worry	0.16	0.12
	Fear of uncertainty	0.17	0.10
	Shyness with strangers	0.13	0.21
	Fatigability and asthenia	0.29	**0.003**

r: Pearson's r.

## Discussion

This study is the first to investigate the relationship between HA and postpartum depression measured by EPDS prospectively in a cohort of pregnant Japanese women. The systematic, longitudinally collected information and subsequent analysis in the current study brings new information regarding the understanding the mental state dynamics of women from pregnancy to postpartum.

We investigated HA levels and depressive state before and after childbirth to assess the role of HA as a risk factor for the development of depressive symptoms prospectively. Moreover, we assessed the levels of HA among mothers that experienced depressive state only after delivery (ie, the TG and CD groups were excluded). We observed different sequences over time regarding the depressive state that might indicate a different etiology of depression during pregnancy and postpartum depression. These differences were not recognized in previous studies, because the previously used postpartum depressive group included women who belonged to the CD group. In addition, as the women included in TG and CD groups may have suffered from mood disorders including MDD or bipolar disorder, we excluded those groups from the current analyses. This exclusion was one of the strengths of our study.

The sample size used in the current study was large and bias effects (ie, recall/reporting bias) were relatively small as prospective design was used in the current study. Moreover, as all subjects were Japanese, genetic and cultural confounders were negligible.

Results demonstrated that there were no differences in mean levels of HA between the ND and PD groups at T1. Thus, our results suggest that HA may not be a significant risk factor for the development of postpartum depression measured by EPDS. Furthermore, our findings indicated that HA may increase according to increase of severity of depressive symptoms (the state dependency of HA) due to significantly positive correlations in changes of EPDS and HA from T1 to T2. In addition, we observed the most significant correlation between EPDS change and Fatigue/Asthenia subscale of HA. Although in order to explore this findings it may be necessary to have additional covariables that could contribute to the association between Fatigue and Asthenia and EPDS, it is of note that the levels of Fatigue and Asthenia may be elevated due to the pregnancy and child birth experience [18]. Therefore, we speculate that this may result in the strongest association between EPDS and Fatigue/Asthenia.

This study had several limitations. First, we evaluated women's mental states only with a self-administered questionnaire. Additionally, histories regarding mood disorders before pregnancy were not assessed, and the ND and PD group also might include people with bipolar disorder [19], [20]. Future studies may find it helpful to assess histories of mood disorders using diagnostic tools such as the SCID in groups with postpartum depression.

In conclusion, this prospective study suggests that high HA, a personality trait, observed during pregnancy may not be a significant risk factor for the development of postpartum depression measured by EPDS. Furthermore, our findings demonstrate that levels of HA may increase according to the onset of depressive symptoms (the state dependency of HA), and decrease as a result of improvement in symptoms of depression. Additional investigations into the state dependency of additional personality traits that are purported to be linked to the onset of MDD are needed.

## Materials and Methods

### Participants

This study was approved by the Ethics Committees of the Nagoya University Graduate School of Medicine and associated institutes and hospitals. Written informed consent was obtained from all participants after the study was described to them in full detail. Participants in this study consisted of women who attended the prenatal program during pregnancy (starting before the 25th week) at two obstetrical hospitals between August 2004 and October 2010. The hospitals were located in the local administrative center of the city of Nagoya (with a population of approximately 2 million people). Participants were randomly selected from the obstetric hospital. Mothers with previous history of mental problems or current treatment for mental problems were excluded from the study, as well as mothers suffering from neonatal pathology, born before 32 weeks of pregnancy. The follow-up period was 6 months after the delivery [10]. Participants were asked to complete self-reported questionnaires about depression and personality (namely, HA traits) at home according to a predetermined schedule.

A total of 647 adults (≥20 years) were recruited for the study. All subjects were Japanese. Several participants were excluded for various reasons including lack of information on age (n=4); incomplete data on HA scores on the TCI (n=7); incomplete EPDS (n=160); and incomplete other data (n=16). Thus, a total of 460 participants (mean age, 31.9±4.2 years; range, 20–44 years) were included.

### Study design

Depressive state (measured by EPDS) and HA were evaluated from early pregnancy to 1 month after postpartum. Depressive state (measured by EPDS) and HA were measured using the EPDS and TCI, respectively. Participants were divided into the following four groups according to severity of depressive symptoms from early pregnancy to 1 month after childbirth as same as our previous study [10]: group 1, non depressive (ND) group (mothers scoring below EPDS threshold in all 3 time points) (n=331); group 2, postpartum depressive (PD) group (mothers scoring above EPDS threshold only at T2) (n=48); group 3, temporary gestational depressive (TG) group (mothers scoring above EPDS threshold only during pregnancy) (n=52); and group 4, continuous depressive (CD) group (mothers scoring above EPDS threshold during both pregnancy and postpartum) (n=29) (Table 1).

EPDS scores obtained during the following three periods were used to classify participants into the four aforementioned groups: early pregnancy (before 25 weeks, T1), late pregnancy (around 36 weeks), and 1 month postpartum (T2). The merit of this classification is to distinguish groups that did not present with symptoms of depression during pregnancy (ND and PD groups) from groups that presented with depressive symptoms during pregnancy (TG and CD groups). Because depressive symptoms were evaluated only at postpartum in most previous studies, the NG and TG groups were combined into a single group and the PD and CD groups were combined into a single group.

Differences in HA scores during T1 were compared between the ND and PD groups to evaluate whether levels of HA in pregnant women served as a risk factor for postpartum depression. The present study did not use structured interviews such as the Structured Clinical Interview for DSM Disorders (SCID) to confirm a history of mood disorders. The TG and CD groups were excluded from these analyses because these groups may include people with mood disorders, including MDD or bipolar disorder. A total of 379 participants (ND, n=331; PD, n=48) were included in these analyses.

Next, the association in the change between EPDS and HA scores was examined in the ND and PD groups to evaluate whether HA levels increased as EPDS increased from T1 to T2, that is, we measured the state dependency of HA. In this analysis we included 99 participants (ND, n=81; PD, n=18; mean age, 32.2±4.1 years; range, 24–44) who submitted HA scores both at T1 and T2. To note, there was a discrepancy in the number of subjects with HA scores between T1 and T2 due to the fact that HA was not assessed during the launch of this cohort study at T2.

### Measures

We investigated the mental state of the participants with two self-administered questionnaires. EPDS is a well-known screening tool for depression in women during pregnancy and postpartum. The TCI is used to assess four dimensions of temperament including HA and three dimensions of character. We examined EPDS and TCI scores during early pregnancy (that is, before 25 weeks at T1) and at 1 month postpartum (at T2).

#### The Edinburgh Postnatal Depression Scale

We evaluated the depressive state (measured by EPDS) of participants during the period right after childbirth using the EPDS [21], [22]. The EPDS is a self-reported questionnaire that includes 10 items designed to screen for postpartum depression in community samples. Each item is scored on a four-point Likert scale (from 0 to 3), with scores ranging from 0 to 30. This scale focuses on the cognitive and affective features of depression, rather than on somatic symptoms. Its sensitivity and specificity in a Japanese community sample were 75% and 93%, respectively, using a cut-off point of 8/9 [23]. The 8/9 cut-off point to screen for depressive women was also used in the present analyses. This questionnaire has also been validated as a screening instrument for use throughout pregnancy and is comparable to other screening scales for depression for use in community samples. When used in community settings, this scale is referred to as the Edinburgh Depression Scale [24].

#### The Temperament and Character Inventory

Personality traits including HA were measured with the TCI. The TCI is a self-reported questionnaire that includes 125 items that tap into four dimensions of temperament (novelty seeking, HA, reward dependence, and persistence) and three dimensions of character (self-directedness, cooperativeness, and self-transcendence). HA was originally assumed to be influenced by the serotonergic system [25]. We used the Japanese version of the TCI-125, which includes 125 questions including 20 items pertaining to HA [26]. HA scores ranged from 0 to 20 and consisted of the following four subscales, anticipatory worry (0–5), fear of uncertainty (0–5), shyness with strangers (0–5), and fatigability and asthenia (0–5).

### Statistical analysis

Analysis of variance (ANOVA) was used to test the mean differences within the four groups divided by EPDS (Table 1). The student t-test was used to compare HA scores between the ND and PD groups at T1 (Table 2). Paired t-test was used to calculate changes in EPDS scores and HA scores between T1 and T2 in the ND and PD group (Table 3 and 4). Cohen's d was used to show the differences in HA and EPDS from T1 to T2 as effect size in the ND and PD group (Table 3 and 4). Cohen's d was calculated from means, standard deviations and sample size in two groups. Correlations between EPDS and HA scores at T1, T2, and changes in EPDS score and HA total score/subscores from T1 to T2 were evaluated based on Pearson's coefficients (r) within the ND+PD group (Table 5). Significance levels were set at p<0.05. All p-values were two-tailed p-values. IBM SPSS Statistics Version 19 (IBM Japan, Tokyo) was used for all analyses.

## Supporting Information

Figure S1 **Changes in EPDS score in ND and PD group.**
(TIF)

Figure S2 **Changes in HA score in ND and PD group.**
(TIF)

Figure S3 **HA and EPDS score changes from T1 to T2 in the ND and PD groups.**
(TIF)
