# Supplementary figures and images for: Prospective Study on the Association between Harm Avoidance and Postpartum Depressive State in a Maternal Cohort of Japanese Women
Source: PLoS One. 2012 Apr 10;7(4):e34725. doi: 10.1371/journal.pone.0034725 (PMC3323560; doi:10.1371/journal.pone.0034725)

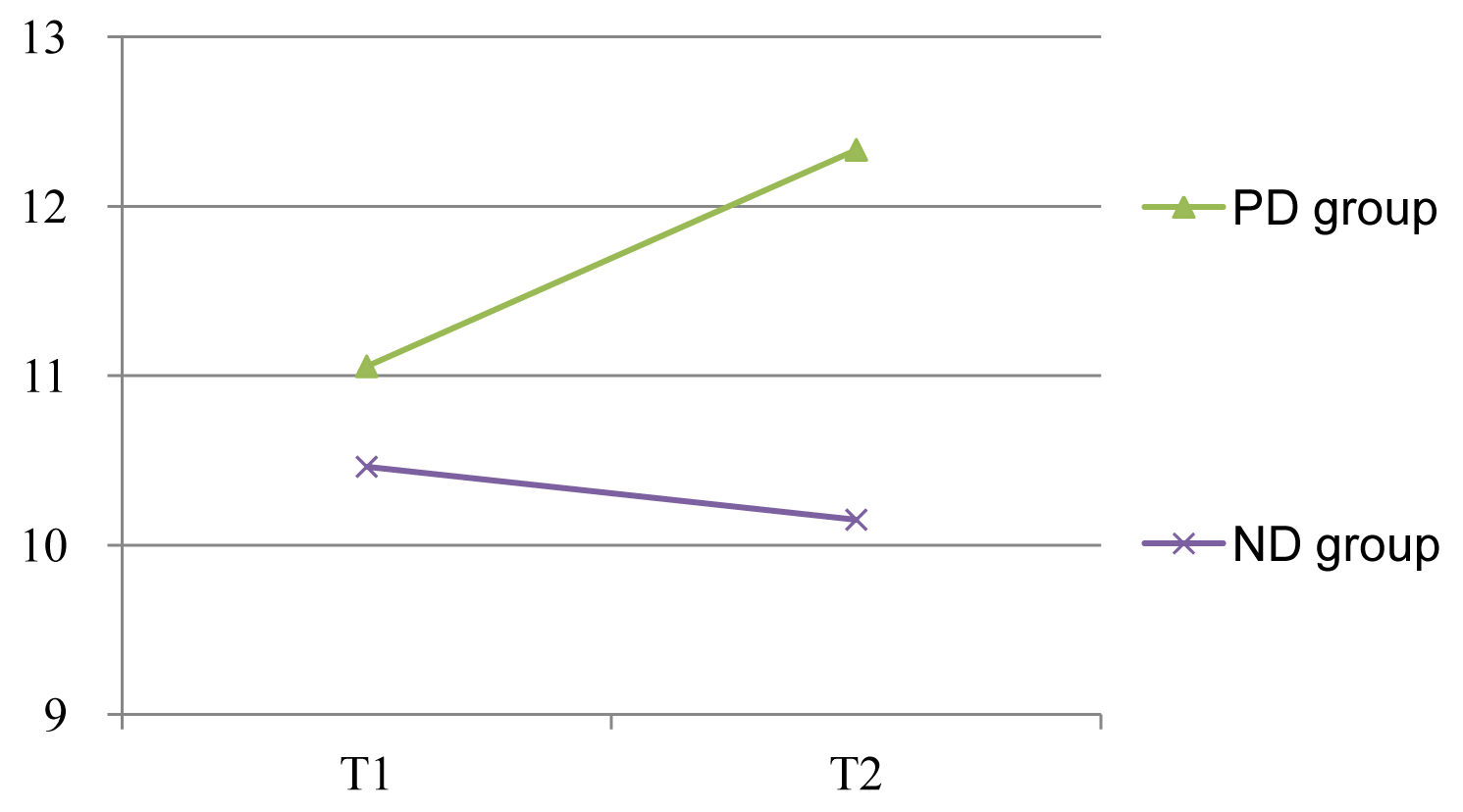

Supplement: Figure S2 — Changes in HA score in ND and PD group. (TIF) [file pone.0034725.s002.tif]

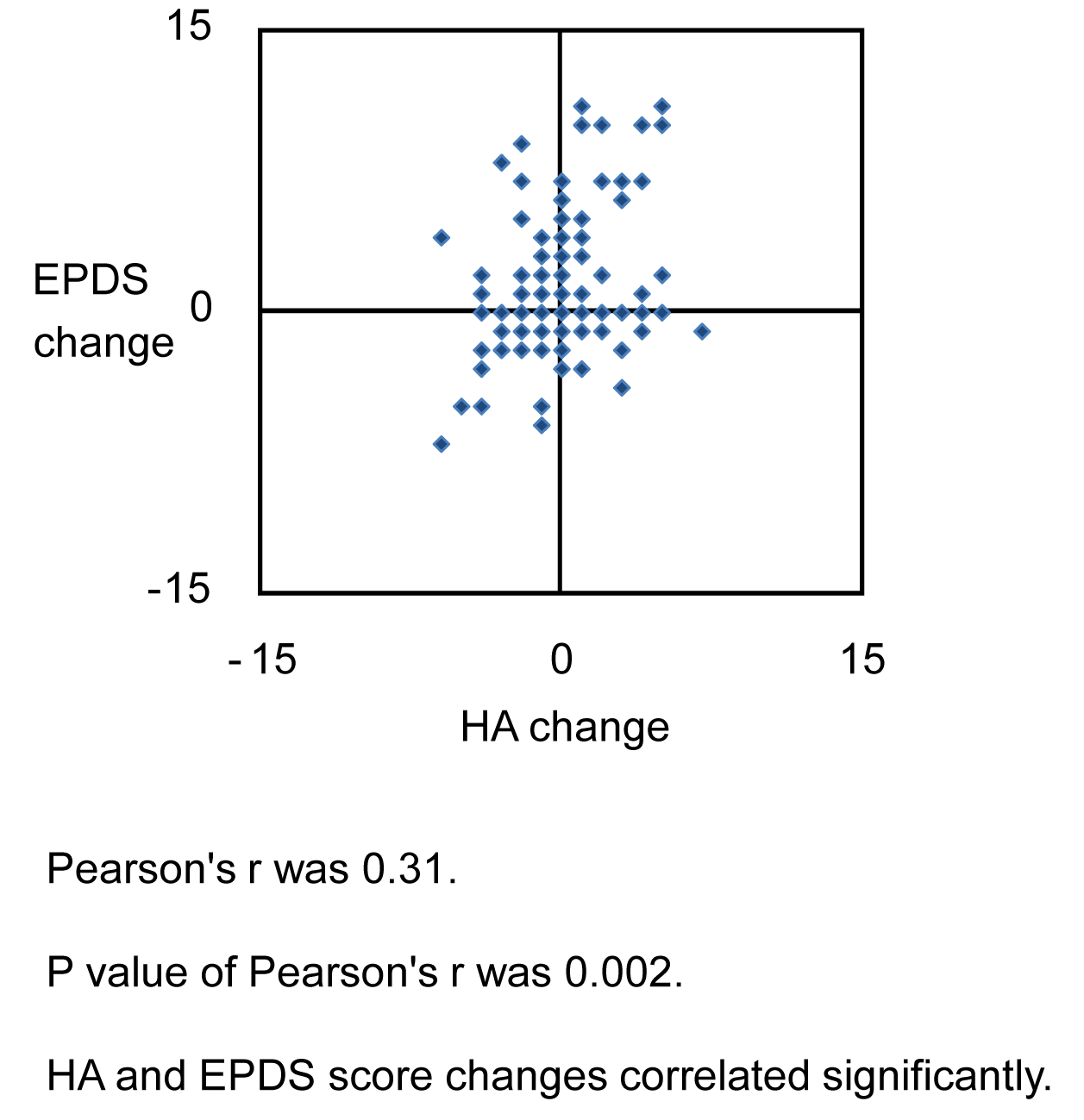

Supplement: Figure S3 — HA and EPDS score changes from T1 to T2 in the ND and PD groups. (TIF) [file pone.0034725.s003.tif]
